# AI-driven prediction of consumer liking of coffee from sensory data

**DOI:** 10.1038/s41538-026-00779-7

**Published:** 2026-03-14

**Authors:** Michael Gunning, Maite Pilar Serantes Laforgue, Jean-Xavier Guinard, Ilias Tagkopoulos

**Affiliations:** 1 Department of Computer Science, University of California, California, CA USA; 2 Genome Center, University of California, California, CA USA; 3 USDA/NSF AI Institute for Next Generation Food Systems (AIFS), University of California, California, CA USA; 4 Department of Food Science and Technology, University of California, California, CA USA; 5 UC Davis Coffee Center, University of California, California, CA USA

**Keywords:** Mathematics and computing, Psychology, Psychology

## Abstract

Understanding and predicting consumer acceptance is critical to commercial success in the coffee industry. This study presents a robust data analysis framework to deconstruct consumer preference using a dataset where 118 consumers rated their liking of 27 black drip coffee samples, the adequacy of select attributes on just-about-right (JAR) scales, and the sensory profile of the coffees with a check-all-that-apply (CATA) task. We integrated four feature-ranking methods to identify key sensory drivers, which informed the development of predictive models to forecast consumer liking. A novel consumer segmentation technique was also introduced, applying k-Means clustering to consumers’ individual preference correlation vectors. JAR acidity, JAR flavor intensity, and CATA sweetness were found to be primary drivers of liking across the population (p-value < 1e-70). The resulting predictive models demonstrated strong performance even with a limited set of 3 sensory features. Consumers were clustered into two segments with contrasting preferences for 12 different sensory attributes. The proposed analytical pipeline provides a comprehensive approach to sensory and consumer data, enabling both the prediction of general consumer liking and the identification of distinct preference segments.

## Introduction

With an estimated 3 billion cups consumed daily, coffee is one of the most widely enjoyed beverages in the world^1^. The success of a coffee product is critically dependent on meeting consumer sensory expectations, which are shaped by a complex interplay of aroma, taste, and mouthfeel. The sensory profile of brewed coffee is influenced by numerous factors, including bean origin, roast level, and brewing parameters such as temperature, grind size, and extraction yield^2,3^. Understanding the key sensory attributes that drive consumer liking is therefore essential for product development and quality control in the coffee industry.

Isolating which specific sensory characteristics lead to consumer acceptance is a tantalizing question. Drivers of liking may also be different for different regions, different coffee species such as Arabica and Robusta, and even different preparation methods. Despite these differences, some drivers of liking for the general coffee-drinking population seem to be consistent, as for example roasted and sweet flavors have been found to be positive influences of consumer liking in various studies, whereas acidity and sourness are often identified as negative drivers of liking^4–6^. But this picture can be flipped for specialty coffee, with darker roasts seen as less desirable, as potentially overwhelming the other flavors in the coffee, and fruitiness, acidity, and light or medium roasts being generally favored by that community^7–9^. In this study, we identify drivers of liking by ranking each sensory attribute’s correlation with respect to liking. Our findings are largely consistent with prior work, with roasted, nutty, and sweet being key positive drivers of liking, and acidity, sourness, and burnt taste being key negative drivers of liking. However, we also show that although highly acidic coffees tend to be disliked, consumers find it almost equally as important to not have too little acidity.

Even with an understanding of which sensory characteristics are preferred by consumers, it is not necessarily straightforward to then recommend a coffee to a consumer. Response surface methodology and preference mapping have been used in previous coffee studies to successfully map product liking and sensory characteristics^5,8,10^. However, even these methods still fail to answer the simple question: given a sample with some set of sensory characteristics, will that sample be liked or disliked? In this research, we tested and evaluated a range of predictive models to predict whether a coffee would be liked or disliked, as well as what the liking score would be for a given coffee.

**Fig. 1:**
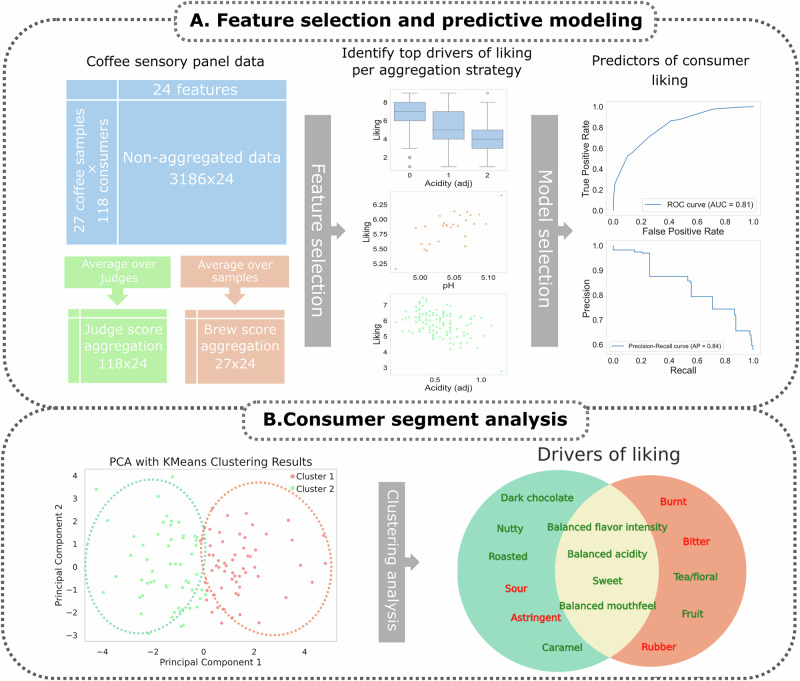
Overview of data aggregation, feature and model selection, and consumer segment analysis. **A** The full dataset of 27 coffee samples evaluated by 118 consumers forming 24 features including sensory and chemical measurements. Feature selection is performed to reveal primary drivers of liking, and then a model selection pipeline is used to build a model to predict liking. **B** 2 consumer segments were identified and analyzed to identify unique and shared drivers of liking.

Of course, different consumers have different preferences. Even when considering consumers within the same region evaluating the same product, it is natural to expect consumer segments with unique preferences^11,12^. Preference mapping is a popular and effective tool to elucidate such segments and is used frequently in food sensory research^13,14^. Bhumiratana et al. identified 6 consumer segments with varying emotional responses to commercial coffee samples^15^. Cotter et al. identified 2 consumer segments on the same coffee dataset used here using preference mapping^8,16^. In this work, a novel consumer clustering method is proposed where individual consumer preference vectors are calculated and compared for similarity. We show that this method can be used to identify novel consumer segments with unique and meaningful sensory preferences. The segments are also analyzed with respect to the brewing parameters and chemical and physical measurements, showing that consumer segments not only have different preferences but also unique perceptions of the same product. We tested the hypothesis that AI can predict consumer liking from sensory data, potentially identifying novel drivers of liking not captured by traditional regression and preference mapping approaches (Fig. 1).

## Results

### Adequacy of flavor intensity (JAR attribute), adequacy of acidity (JAR attribute), and sweetness (CATA attribute) are key drivers of consumer liking of coffee

Before data aggregation, all three JAR attributes (i.e., flavor intensity, acidity and mouthfeel) were found to be highly correlated with consumer liking, in particular adequacy of flavor intensity (Spearman: −0.45, *p*-value: 1.84e–156) and adequacy of acidity (Spearman: −0.44, *p*-value: 4.04e–150) were ranked as the two most important features with respect to consumer liking. JAR features combine a consumer’s perception of attribute strength and appropriateness, so it is not surprising to see a strong relationship with hedonic acceptance^17^. In the case of JAR features, note that a negative correlation with hedonic liking indicates that high deviations from what a consumer views as “adequate” tend to be given lower liking scores. Adequacy of mouthfeel was found to be less important, but still among the top 10 features by average rank. Notably, there were significant differences between liking scores (Kruskal–Wallis test *p*-value: <0.001) for coffees rated as having too much flavor intensity and too little flavor intensity. However, for adequacy of acidity, both extremes performed almost identically with respect to liking. In terms of CATA features, sweetness was found to be the most important and third most important overall, followed by dark chocolate and nutty. For the non-aggregated dataset, only sensory features were found to be good indicators of liking. Surprisingly, no significant correlation was observed between liking and any of the chemical measurements, physical measurements, and brewing parameters.

Within the brew score aggregation dataset, different trends emerge; in particular, JAR scores all drop in importance. This is not surprising as these scores are based on each consumer’s subjective opinions of each sample, so a sample that is rated as “too acidic” by one consumer might be “just about right” to another^18^. Only JAR acidity was found to have a statistically significant correlation with liking. Differences in CATA attributes are present as well; the top five CATA attributes for this aggregation strategy were nutty, roasted, green vegetable, rubber, and citrus. Notably, the pH of each sample was found to be the best indicator of liking for this aggregation strategy, with higher pH coffees tending to be more well-liked than lower pH coffees. This finding is consistent with attributes usually associated with more acidic coffees, such as citrus and sour appearing to be generally disliked by the consumers in this study.

**Fig. 2:**
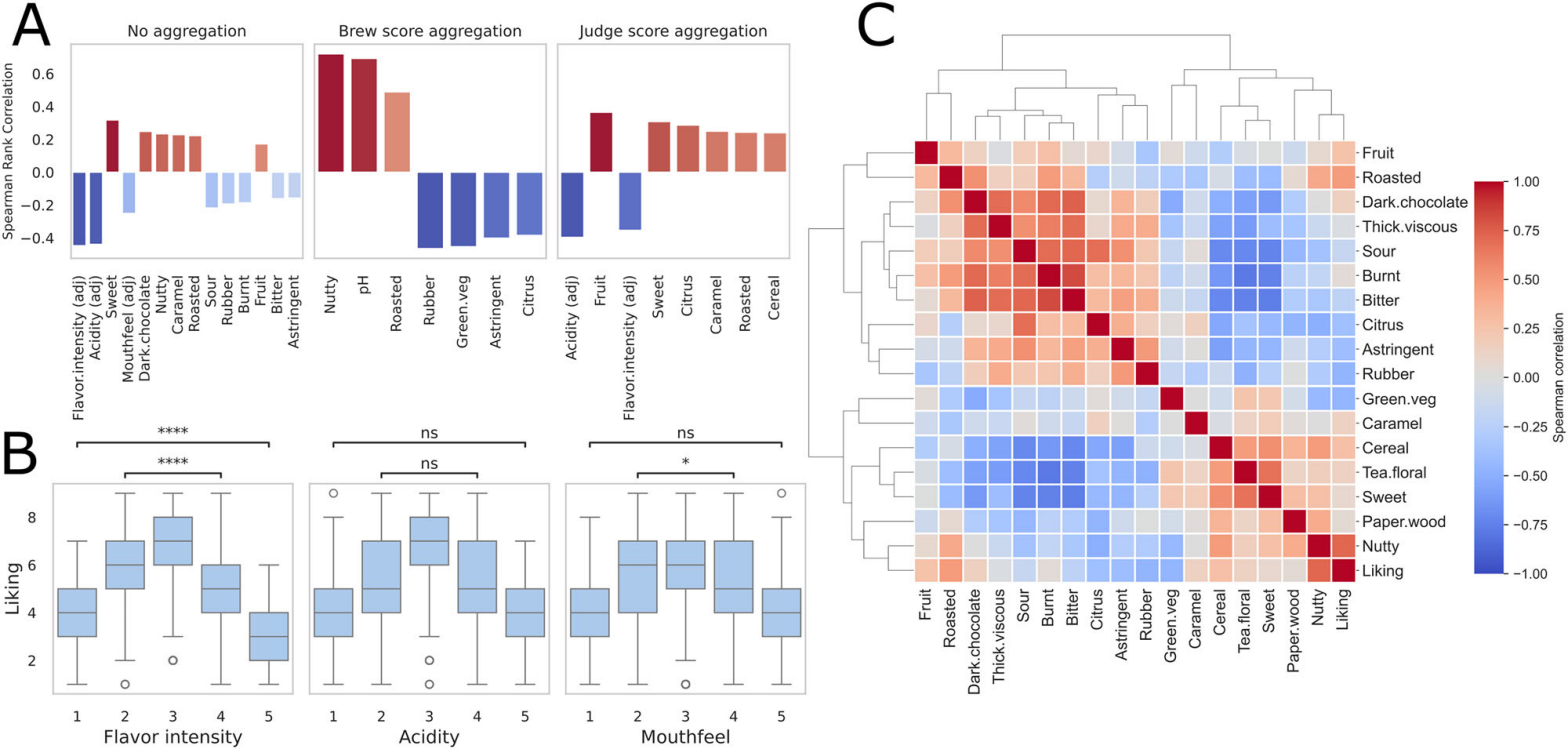
Sensory variable correlation with liking and other sensory variables. **A** Spearman rank correlation between coffee liking and different flavor attributes under three data aggregation schemes. **B** Analysis of JAR features. Values closer to 3 (“just about right”) are associated with high liking scores for all features, but analysis of flavor intensity revealed that consumers are more accepting of coffee with too low flavor intensity than too high. **C** Heatmap showing correlation among sensory variables across samples.

The consumer score aggregation strategy essentially condenses the dataset into 118 ratings for a single sample of coffee. Consumers who thought the set of samples overall was “sweet” tended to like the set of samples overall. Likewise, consumers who gave more JAR ratings outside of the “just about right” range were generally less satisfied by the coffees tested. Similar to the non-aggregated data, the JAR attributes acidity and flavor intensity were the most important drivers of liking. Mouthfeel on the other hand, was considerably less important and had no statistically significant correlation with liking. Although sweet was once again identified as an important attribute, the other top CATA attributes were fruit, citrus, and cereal, which were notably less important within the other aggregation strategies. A comparison of the top, statistically significant (*p*-value < 0.05) drivers of liking for each aggregation strategy is presented in Fig. 2.

After clustering, a similar analysis can be done to determine the drivers of liking per cluster. For both clusters, the JAR attributes “flavor intensity” and “acidity” were the top drivers of liking, although for cluster A, acidity was more important, and for cluster B, flavor intensity was at the top. For cluster A, the top 5 CATA attributes were dark chocolate, sweet, roasted, nutty, and caramel. Compared to cluster B, for which the top CATA attributes were sweet, fruit, burnt (negatively correlated), tea-floral, and bitter (negatively correlated). Among these top attributes, only ‘sweet’ was shared between them. pH was the only physical or chemical measurement relevant to the per-cluster drivers of liking, which, for cluster A, was positively correlated with liking (13th highest rated feature for cluster A). Brewing parameters were not significantly aligned with liking for either cluster.

### The consumer liking of black drip coffee can be predicted accurately with just 3 sensory measures

When trained on the non-aggregated dataset, the predictive model demonstrated moderate performance. The binary classifier model trained on flavor intensity (JAR), acidity (JAR), and sweet (CATA) was able to predict if a consumer would like or dislike a coffee with an accuracy of 0.75 on the holdout set. The model’s precision, recall, F1 score, and AUROC score were 0.74, 0.86, 0.80, and 0.81, respectively. These results are compared with a majority classifier as a baseline (Table 1).

**Table 1:** Final model holdout set performance compared to baseline

Method	Accuracy	Precision	Recall	F1 Score	AUROC	Specificity
**Majority classifier**	0.58	0.58	1	0.73	0.5	0
**AdaBoost**	0.75	0.74	0.86	0.80	0.81	0.59

For the regression model, cross-validation (5-fold) revealed an average R^2^ score of 0.47, an average Mean Squared Error (MSE) of 1.59, and an average Mean Absolute Error (MAE) of 1.01. When evaluated on an independent holdout set, the model exhibited an R^2^ score of 0.47. The holdout set MAE and MSE were 1.03 and 1.64, respectively, consistent with the cross-validation results. These metrics suggest that the model provides reasonably accurate predictions of consumer liking, with an average absolute prediction error of approximately one liking unit on the 9-point hedonic scale.

A separate model was trained on the data after aggregating by coffee brew sample. Training a predictive model with this aggregation strategy is difficult due to the small number of samples to train from, only 27. We performed nested leave-one-out cross-validation and present here only the results from the outer cross-validation, as there were not enough data to generate a holdout set. The top performing regressor, a random forest, had an aggregated R^2^ score of 0.40 on the out-of-sample predictions. The MSE was 0.04, and the MAE was 0.15. A binary classification model was also evaluated in the same manner, achieving an accuracy of 0.81, an F1 score of 0.87, a precision of 0.85, and a recall of 0.89.

**Fig. 3:**
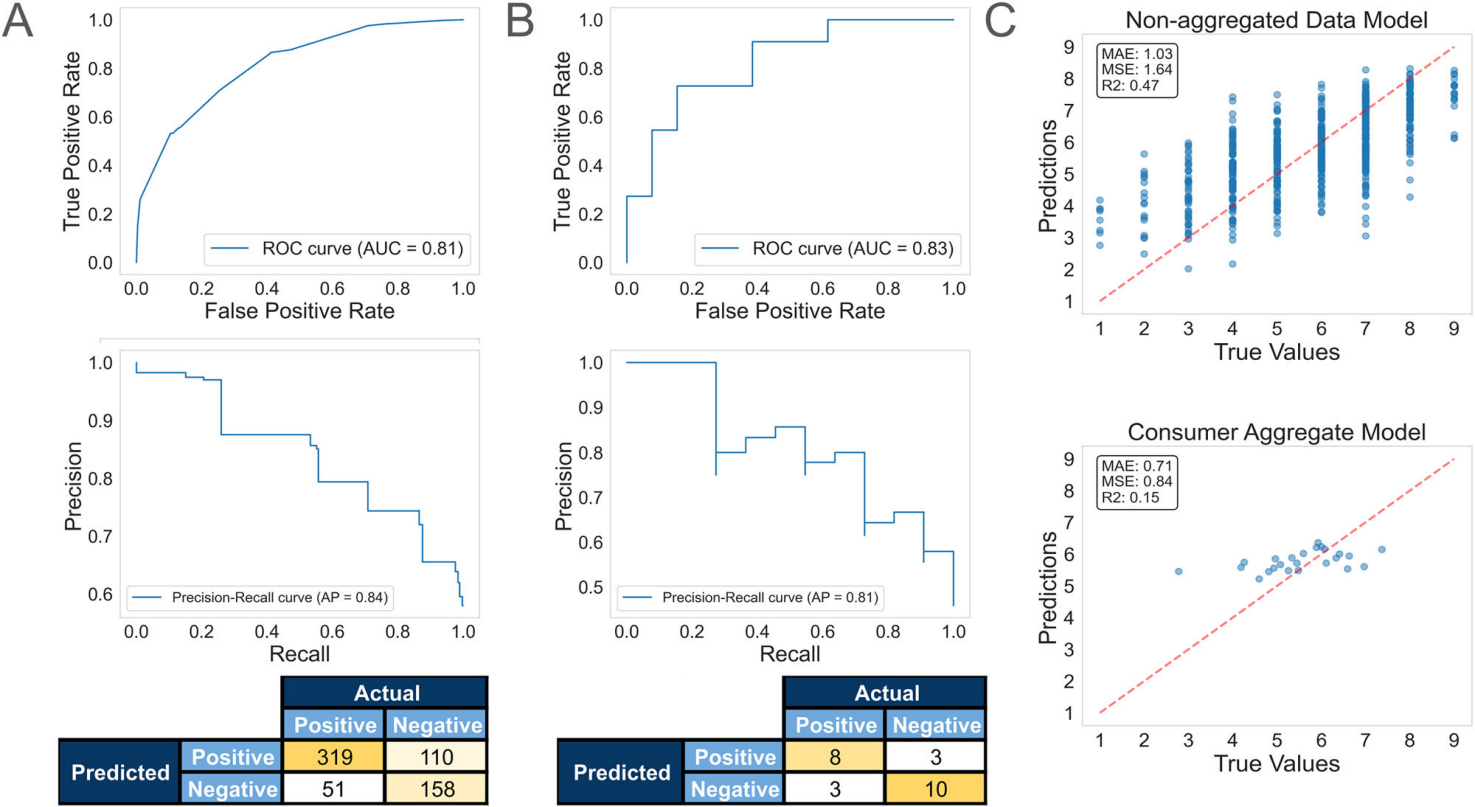
Predictive model results. **A** Non-aggregated dataset binary classifier ROC curve, PR curve, and confusion matrix. **B** Consumer score aggregation binary classifier ROC curve, PR curve, and confusion matrix. **C** Regression results for non-aggregated data and consumer score aggregation.

Aggregating by consumer also leads to a viable predictive model, but again, due to the reduced number of total samples, the results are limited. Five-fold cross-validation performance showed an average R^2^ score of 0.28, an MSE of 0.33, and an MAE of 0.48. The holdout set performance was slightly worse with an R^2^ score of 0.15, MSE of 0.84, and an MAE of 0.71, but all metrics were within a standard deviation of cross-validation performance. A classifier was also trained and achieved results similar to the non-aggregated dataset classifier, with an accuracy of 0.75, an F1 score of 0.73, an AUROC of 0.83, and a precision and recall of 0.73. Figure 3 compares the classification and regression results for the consumer aggregated data and non-aggregated data. The brew aggregate results are excluded, as it was not possible to create a large enough independent test set. The cross-validation results for all models can be found in Tables 2 and 3.

**Table 2:** Cross-validation performance of best classification model for each data aggregation strategy

Data	Classifier	Outlier detection	F1 Score	Accuracy	Precision	Recall
**Non-aggregated data**	AdaBoost	none	0.80 ± 0.01	0.74 ± 0.01	0.75 ± 0.01	0.86 ± 0.02
**Judge score aggregation**	Naïve Bayes	LOF	0.72 ± 0.09	0.69 ± 0.1	0.72 ± 0.1	0.75 ± 0.17
**Brew score aggregation**	AdaBoost	none	0.87	0.81	0.85	0.89
**Brew parameters only**	AdaBoost	LOF	0.89	0.85	0.89	0.89

**Table 3:** Cross-validation performance of the best regression model for each data aggregation strategy

Data	Regressor	Outlier detection	R2 Score	Mean squared error	Mean absolute error
**Non-aggregated data**	MLP	none	0.47 ± 0.01	1.59 ± 0.10	1.01 ± 0.03
**Judge score aggregation**	Random forest	none	0.28 ± 0.16	0.33 ± 0.12	0.48 ± 0.10
**Brew score aggregation**	Random forest	LOF	0.41	0.04	0.15
**Brew parameters only**	AdaBoost	LOF	0.37	0.04	0.16

### Liking prediction is possible using only brew parameters and chemical measurements

Chemical measurements and brewing parameters are generally easier to affect than sensory characteristics, as they rely on objective measurements rather than subjective consumer opinions. Recursive feature elimination was performed on only this subset of features, and then a model was trained based on the brew-aggregated dataset. As these features are static with respect to the 27 different coffee brews tested, this aggregation had to be used. Given the small size of the dataset post-aggregation, an independent test set could not be used for final evaluation. Instead, the results are from the leave-one-out cross-validation performance, which was chosen as it is a robust method of evaluating predictions on small datasets^19^. To provide an unbiased estimate of model performance, as with the previous brew-aggregate model, nested cross-validation was employed, and the results presented here are the outer-CV performance. The features selected for the final model were total dissolved solids, pH, pour temperature, and “volume,” referring to the volume of 0.1 molar NaOH added to titrate 50 mL of brewed coffee to a target pH of 8.2. Performance of the final model was reasonably strong, with an accuracy of 0.78, an F1 score of 0.84, and precision and recall both 0.84. A regressor was trained on the same features and achieved an R^2^ score of 0.24, a MSE of 0.05, a noticeable drop compared to the model trained with sensory features.

### Consumer segmentation reveals diversity in consumer preferences and even consumer sensory perception

Two clusters of consumers were identified with our novel approach to preference clustering, size *n* = 57 and *n* = 61. Twelve (12) features were evaluated by the Kruskal–Wallis test as having statistically significant (*p*-value < 0.05) differences between the clusters. The significant features are listed in Table 4, as well as the average of each feature per cluster (Table 4). We compare these clusters to the two consumer segments identified by Cotter et al. using a hierarchical cluster analysis with Ward’s method^8^. Their results are similar, but with some key differences. They found that one cluster had a strong preference for coffees with “tea-floral,” “sweet,” and “cereal” notes while the other cluster preferred “roasted,” “dark chocolate,” and “nutty” coffees. Similar trends can be found in the clusters identified here, where there is clearly a stronger preference by cluster A for “dark chocolate,” “roasted,” and “caramel,” and a stronger preference by cluster B for “tea-floral” and “fruit”. Despite these similarities, the overlap between consumers in the similar clusters is only about 25% for the “roasted” clusters and 34% for the “fruity” clusters. While our clustering method found 12 features with statistically significant differences, the Cotter et al. method only identified 5 (Kruskal–Wallis test, *p*-value < 0.05). On the other hand, the Cotter et al. clusters better separated consumers based on preferred coffee brews, with 10 brews having a statistically significant difference in liking, compared to our clusters, where only two coffees exhibited a significant difference in liking between clusters (Supplementary Figs. S1, S2).

**Table 4:** Average cluster affinity for significantly different attributes

Cluster	Tea, floral (1.44E-09)	Dark chocolate (1.71E-08)	Citrus (4.69E-08)	Roasted (2.21E-07)	Nutty (2.91E-06)	Caramel (9.29E-05)
A	−0.03	0.36	−0.08	0.34	0.35	0.25
B	0.22	0.14	0.14	0.10	0.13	0.11
**Cluster**	**Acidity** **(1.45E-04)**	**Bitter** **(1.11E-03)**	**Sour** (**1.20E-03)**	**Burnt** **(1.58E-03)**	**Fruit** **(1.83E-03)**	**Cereal** **(0.04)**
A	−0.52	−0.09	−0.27	−0.11	0.06	0.06
B	−0.38	−0.23	−0.13	−0.25	0.18	0.00

**Fig. 4:**
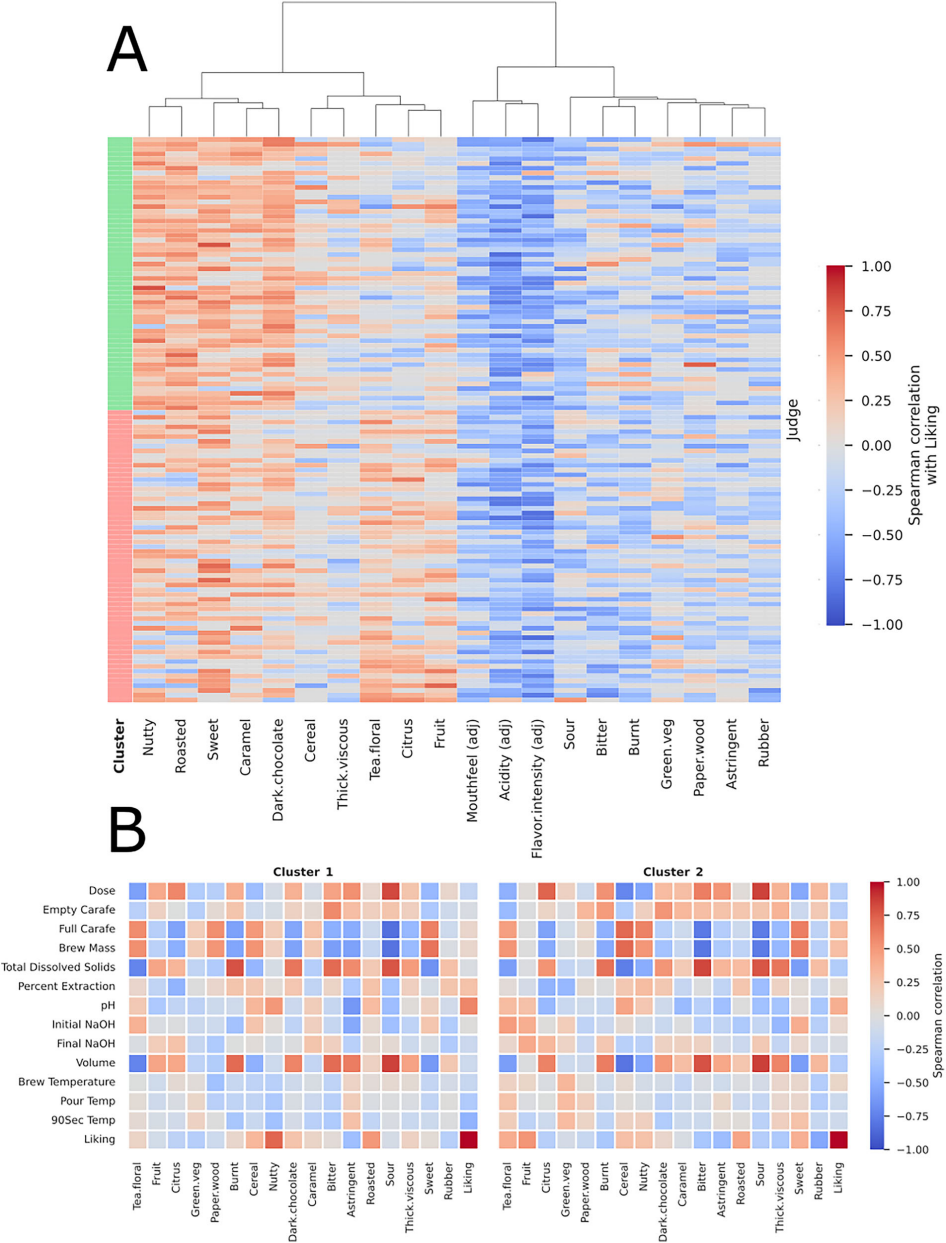
Cluster analysis. **A** Heatmap of Spearman correlation scores between sensory attributes and liking per consumer. **B** Heatmaps of each cluster’s Spearman correlation between sensory attributes and coffee brewing parameters.

Figure 4A shows the association between liking scores and sensory attributes per consumer. It is clear that some attributes are perceived more positively and some more negatively overall. Nutty, roasted, sweet, dark chocolate, and caramel were positively correlated with liking for almost all consumers. Likewise, sour, bitter, burnt, green vegetables, paper/wood, astringent, and rubber were negatively correlated with liking for the majority of consumers. It is also clear how the attributes are perceived by the clusters. Higher liking correlations for attributes like dark chocolate and roasted can be observed for cluster 1, whereas cluster 2 has higher correlations for tea-floral, citrus, and fruit.

We also examine the role of the coffee physical and chemical parameters with respect to the sensory attributes. Figure 4B shows the per-cluster association between sensory attributes and coffee measurements. Interestingly, the sensory perceptions associated with the brewing parameters were found to be different between the clusters. For example, cluster 1 associated higher TDS coffees with the “fruit” sensory attribute, while cluster 2 did not. The Spearman correlation between TDS and “fruit” for cluster 1 was 0.44 (*p*-value: 0.02), but for cluster 2, there was no significant correlation between these features. Similarly, consumers in cluster 1 tended to experience coffees with a higher pH as “nutty” (Spearman 0.51, *p*-value: 0.01) while there was no clear association between these variables for cluster 2 (Spearman 0.23*, p*-value: 0.25).

Finally, per-cluster models were attempted, but the results were limited. While a regression model trained on only the consumers in cluster 2 showed improved performance (*R*^2^ = 0.54) compared to performance on the full dataset (*R*^2^ = 0.47), a model trained on cluster 1 had a significant drop in performance (*R*^2^ = 0.38). Even when considering that the per-cluster models were trained on data from less consumers, the average performance of both per-cluster models was only moderately better than a non-clustered model (Supplementary Fig. S3).

## Discussion

Analyzing what influences consumer perception and acceptance of coffee is of key interest to the coffee industry. The question we asked with our analysis of Cotter et al.’s data is whether AI would shed a new or different light on the identification of sensory drivers of liking or the prediction of consumer liking based on sensory data. If we compare our machine learning approach to the traditional sensometrics employed by Guinard et al.^16^, to design the new Brewing Control Chart, they derived from descriptive analysis studies^20,21^ and the Cotter et al. consumer research^8^, we can state that there are some commonalities as well as some differences. Guinard et al.^16^, identified two preference segments (somewhat different in their composition and preferences from the ones we uncovered in our analysis—granted, with a different approach) for whom the main drivers of liking were sweetness and black tea (+) and acid/sour, astringent, bitter, roasted and viscous/thick (-) for their first preference segment, and two contrasting profiles of berry, citrus, dried fruit, acid/sour (+), and bitter, roasted, black tea (-) or roasted, black tea (+) and acid/sour, citrus, berry and sweet (-). Their list of positive and negative drivers of liking, therefore, included the taste attributes of sweetness, bitterness, and acidity/sourness, the aromatic notes of berry, citrus, dried fruit, roasted character, and black tea, and the trigeminal characteristics of astringency, viscosity, and thickness. While our AI approach did identify some of those attributes as key drivers of liking for our two consumer segments, chief among them sweetness, it also found dark chocolate and nutty characters and pH to be significant drivers of liking. But most importantly, it enabled the prediction of consumer liking from 3 sensory measures only—JAR acidity, JAR flavor intensity, and CATA sweetness.

That JAR attributes would be good predictors of liking is not surprising because of the hybrid nature of JAR scaling as a construct that combines intensity and hedonic aspects in judging the adequacy of an attribute. Sweetness, on the other hand, is more surprising. Not because sweet taste is not an innately pleasant quality, it is! But because sweet sugars are present in coffee at concentrations below human detection thresholds^22,23^, such that this “sweetness” more likely refers to an aromatic or flavor concept of “brown” (i.e., caramel) or “fruity” (citrus, berries) sweetness than the actual sweet taste^24^; or that it is the absence of bitterness and acidity that makes the coffee taste “sweet”. We also show that certain sensory characteristics have highly positive or negative correlations between one another, showing that sweetness is positively correlated with tea-floral and cereal, and negatively correlated with the burnt, bitter, and sour attributes, which may serve to better explain how consumers experience sweetness in black coffee (Fig. 2).

Sensory perceptions are susceptible to small changes in how coffee is brewed. Of the three brew parameters adjusted in this dataset, “total dissolved solids” was found to have the highest impact on flavor perception and consumer liking, followed by percent extraction. Brewing temperature was not found to impact the sensory profile of the coffees or the overall consumer liking, consistent with prior work^8,21,25^.

Notably, consumers seem to be more accepting of coffees with too little flavor intensity compared to too much flavor intensity. Given the strong relationship identified between TDS and flavor intensity, it may be better to err on the side of less TDS and therefore less flavor intensity if the goal is to appeal to the widest range of consumers. Samples rated as too acidic were generally disliked (Fig. 2) and were often described as “sour,” a strong negative driver of liking. Other research has shown similar findings, that too much acidity should be avoided, and a sour taste in coffee tends to be disliked^8,15,26^. Our findings indicated that samples with too little acidity were strongly disliked as well. Therefore, *striking the right* acidity rather than *reducing* acidity is critical to producing well-liked coffees, highlighting the often-challenging management of sensory characteristics. These findings were consistent across consumer segments (Supplementary Fig. S4), indicating that these preferences appear to be consistent among consumers.

Prior work by Cotter et al. using the dataset they produced, and we analyze here, identified two distinct consumer clusters of similar size, which we will compare to our own findings. Cotter et al. used Ward linkage hierarchical clustering on the covariance matrix of normalized consumer liking scores per coffee^8^. The clusters identified with this method show a strong separation between preferred coffees per cluster, but do not distinguish as strongly between preferred sensory characteristics per cluster (Supplementary Figs. S1, S2). The method presented in this paper incorporates both hedonic liking as well as sensory characteristics, so these differences are not unexpected but highlight how our clustering provides a different view of consumer segments. Our cluster analysis also revealed differences in consumer perception of the same physical attributes. Although this dataset contained no background information about the consumers, future work could examine the role that demographics, coffee drinking habits, and even genetics may play in explaining these differences in perception, all three of which have been found to play a role in consumer coffee preferences^27,28^.

Moreover, we have provided a more general analysis of how these sensory attributes contribute to the overall consumer liking of the coffee samples. Although only a single variety of coffee was evaluated, strong correlations with liking were observed with the key drivers of liking we identified. Therefore, it seems likely that the correct acidity, most likely middle of the typical range for coffee, the correct flavor intensity, and sweetness would be important to consumers across a variety of coffees. The feature and model selection pipeline presented in this analysis can be used in future sensory research for identifying drivers of liking and predicting liking given sensory features. While sensory properties traditionally are measured by descriptive analysis with trained judges, we feel there is some merit to using CATA selections by consumers for the purpose of characterizing the sensory properties of a set of products, for the purpose of predicting liking, because the selection of each consumer and his/her liking can be used to train the models. Given a reasonably large dataset of CATA sensory characteristics (or descriptive analysis ratings) and liking scores, building and evaluating a predictive model should be straightforward.

While our analysis provides a robust methodology for future sensory research, its findings are based on a single coffee variety. Future work should therefore focus on applying this framework to diverse coffee types and consumer populations to build a more universal understanding of coffee preference. Because Cotter et al. worked with variations of a medium roast coffee, there was no opportunity to investigate the role of roasting in predicting consumer acceptance here, yet it often is a key driver of consumer liking^29^. Indeed, it is difficult to estimate how these findings might generalize to a wider variety of coffees and consumers. The dataset used was collected exclusively from coffee drinkers in California, so these findings may not be applicable to other populations. In general, more work is needed in applying and evaluating machine learning-based predictors to sensory data.

This study successfully developed a comprehensive AI/machine learning framework for analyzing consumer sensory data to understand the drivers of liking for black drip coffee. We identified that adequate (JAR) acidity, adequate (JAR) flavor intensity, and (CATA) sweetness were critical attributes influencing consumer preference. Using this knowledge, we demonstrated that predictive models can accurately predict consumer liking from a small number of sensory inputs. Our clustering method also proved effective, identifying two distinct consumer segments with unique preferences for roasted/chocolate versus fruity/floral notes, who also perceived the effects of brewing parameters differently.

## METHODS

### Dataset overview

The dataset “Consumer preference data for black coffee” consists of consumer hedonic ratings for 27 samples of a coffee prepared with varying brewing parameters. Originally collected by Cotter et al., the study consisted of 118 consumers who evaluated each of the 27 samples for 3186 total rows in the dataset^8^. The coffee samples were varied based on 3 brew temperatures, 3 levels of brew strength measured as total dissolved solids (TDS), and 3 levels of extraction yield measured as percent extraction (PE), yielding 27 unique brews that form a full factorial combination of the three brewing parameters. One hundred and eighteen (118) black coffee consumers evaluated each coffee for liking on the 9-point hedonic scale^30,31^, the adequacy of 3 attributes (flavor intensity, acidity and mouthfeel) on just-about-right (JAR) scales, the sensory attributes of the coffee from a check-all-that-apply (CATA) list of 17 attributes, as well as purchase intent at a $3 price point on a 5-point purchase intent scale. Finally, brewing parameters (coffee maker setting, grind size, etc.) as well as physical and chemical measurements (pH, TDS, temperature, etc.) are included as 13 continuous features and 5 categorical features^8,31^.

### Data aggregation strategies

We compare several aggregation strategies of the dataset, which we will refer to as the non-aggregated data, brew score aggregation, and consumer score aggregation. The non-aggregated data is simply the full dataset, with all 3186 total rows. Brew score aggregation transforms the dataset by taking the mean of each feature across the 118 consumer ratings of each coffee sample, yielding 27 rows representing the 27 coffee brews in the dataset. This strategy can identify trends in liking with respect to the individual coffee brews. Consumer score aggregation likewise takes the mean of each feature across the 27 brews, yielding a dataset of 118 rows. Aggregating this way shows trends that apply across all samples and may be a better indicator of what impacts consumer liking of coffee in general. Refer to Fig. 1A for an overview of these strategies.

### Drivers of liking analysis

To identify the drivers of liking for coffee, each feature in the dataset was evaluated and ranked with respect to its impact on consumer liking. To account for the inherent non-linear, non-monotonic relationship between JAR features and hedonic liking, adjusted JAR scores were calculated by centering the score around 0 and then taking the absolute value. The result is a score that captures the JAR feature’s perceived adequacy, with 2 being just-about-right, and lower values indicating a perception of either lacking or excessive intensity. Although some information is lost following this transformation, this allows for a direct analysis of each JAR feature’s correlation with liking, as well as allows for better comparability among features. Each feature was ranked by its Pearson correlation, Spearman correlation, and mutual information with respect to liking. Recursive feature elimination (RFE) using a gradient-boosting regressor model was also performed to generate an additional ranking of each feature. Recursive feature elimination is a procedure in which a model is iteratively trained with smaller subsets of features, at each step removing the least important feature^32^. Finally, an average ranking was calculated based on each feature’s 4 rankings with respect to hedonic liking. This average ranking was used to identify the top drivers of liking for each data aggregation strategy, as well as sort the features for later sequential feature selection.

### Predictors of consumer liking based on sensory variables

Predictive models were trained for each of the three data aggregation strategies, as well as for a dataset containing only the brewing parameters and chemical measurements of the coffees. A regression model and binary classifier were trained for each case, yielding 8 total final models. To generate binary liking ratings, a liking score of 6 or greater on the 9-point hedonic scale was chosen as the cut-off for the target class, as this score indicates that the sample was at least “somewhat liked” on the scale. Five (5) indicates that a sample was “neither liked nor disliked,” but it is likely more useful to know if a potential consumer will actually like a sample, rather than if the sample is just “not-disliked” ^30,31^. Moreover, a cutoff at 6 rather than 5 led to a more even split in the data: 61% liked/39% disliked with a cutoff at 6, compared to 75% liked/25% disliked with a cutoff at 5. For models trained on an aggregated dataset, a sample is considered “liked” if it is above the average liking for that aggregation strategy.

Regression and binary classification models were tested for each data aggregation strategy, and for each case, 5 different models were compared and evaluated. The models tested include support vector machine (SVM), naïve bayes (NB), AdaBoost, multilayer perceptron (MLP), and random forest (RF)^33–37^. The dataset was split into training (80%) and testing (20%) sets. Grid search was performed on the training set to select each model, hyperparameters, data scaling method, and outlier detection strategy. Local outlier factor (LOF) and isolation forest were both tested as outlier detection methods^38,39^. For all but the brew-aggregated models, 5-fold cross-validation was used to compare different model/hyperparameter selections, and then each model was evaluated on a final holdout set, consisting of 20% of the original data not seen by the model previously. As only 27 unique coffee brews were evaluated, when the dataset is aggregated per brew, there is not enough data to have a meaningful holdout set. In these cases, nested cross-validation was performed, and only the outer cross-validation results are presented. Leave-one-out cross-validation was used for these models as the size of the dataset was small, and this method provides the most robust possible evaluation given the small number of samples^19^.

Sequential feature selection was performed based on the previously calculated average rank of each feature. A model was tested and evaluated using 5-fold cross-validation performance, a gradient-boosting regressor for the regression models, and a random forest classifier for the classification model. Features were added sequentially in order of their average rank, and the model’s performance was assessed at each increment. The final number of features was then selected to achieve an optimal balance between the total number of features and overall model performance.

### Consumer segmentation

To understand individual drivers of liking, the Spearman correlation between consumer liking and each CATA and JAR feature was calculated for each consumer. This allows for the identification of features that significantly influence an *individual* consumer’s liking (e.g., a high correlation between “roasted” and liking for a specific consumer suggests “roasted” is an important attribute for that consumer). Each consumer is then represented as a 17-dimensional vector, where each component corresponds to the consumer’s liking of a particular sensory attribute. Before clustering, the vectors were z-score normalized, and principal component analysis was used for dimensionality reduction. The elbow method was used to identify the number of components to keep, in this case 7 (Supplementary Figure S5)^40^. k-Means clustering was used to identify groups of these consumer sensory vectors^41^. To identify an optimal k, we compared the performance of k-Means with different numbers of clusters in terms of distortion score, silhouette score, Calinski-Harabasz index, and Bayesian information criterion^42–44^. *k* = 2 was chosen as it was optimal across most metrics and was most comparable to the Cotter et al. clusters, wherein 2 clusters of consumers were also identified (Supplementary Fig. S5). This approach assumes that consumers with similar preferences will exhibit more closely aligned feature-liking correlation vectors compared to consumers with dissimilar preferences. This clustering strategy attempts to maximize the differences in individual consumer sensory preferences between or among clusters. Using this method, two consumer clusters were identified as having statistically significant differences in preferences for 12 sensory attributes. Additionally, we trained separate predictive models on only the consumers within each segment.

## Supplementary information

Supplementary materials

Supplementary_feature_stats

Supplementary_grid_search_results

## Data Availability

The dataset used in this study was sourced from and is available at: https://datadryad.org/dataset/doi:10.25338/B8993H.

## Code availability

All code and instructions on how to reproduce the results can be found at https://github.com/mhgun/sensory_prediction.

## References

[1] “Coffee Year 2023/24,” International Coffee Organization, Sep. 2024. Accessed: Sep. 09, 2025. [Online]. Available: https://www.ico.org/documents/cy2024-25/annual-review-2023-2024-e.pdf.

[2] Seninde, D. R. & Chambers, E. Coffee flavor: a review. *Beverages* **6**, 44, 10.3390/beverages6030044 (2020).

[3] Sunarharum, W. B., Williams, D. J. & Smyth, H. E. Complexity of coffee flavor: a compositional and sensory perspective. *Food Res. Int.* **62**, 315–325, 10.1016/j.foodres.2014.02.030 (2014).

[4] Geel, L., Kinnear, M. & de Kock, H. L. Relating consumer preferences to sensory attributes of instant coffee. *Food Qual. Prefer.* **16**, 237–244, 10.1016/j.foodqual.2004.04.014 (2005).

[5] Condelli, N. et al. Drivers of coffee liking: effect of physicochemical characteristics and aromatic profile on consumers’ acceptability of mono-origin and mono-variety coffees. *J. Food Sci.* **87**, 4688–4702, 10.1111/1750-3841.16323 (2022). 36112567 10.1111/1750-3841.16323 PMC9826037

[6] Roman-Maldonado, Y., Gutiérrez-Salomón, A. L., Jaimez-Ordaz, J., García-Barrón, S. E. & Barajas-Ramírez, J. A. Drivers of liking to predict consumers’ acceptance of local coffee from indigenous Mexican regions. *Eur. Food Res. Technol.* **248**, 467–475 (2022).

[7] Spencer, M., Sage, E., Velez, M. & Guinard, J.-X. Using single free sorting and multivariate exploratory methods to design a new coffee taster’s flavor wheel. *J. Food Sci.* **81**, S2997–S3005 (2016). 27861864 10.1111/1750-3841.13555 PMC5215420

[8] Cotter, A. R., Batali, M. E., Ristenpart, W. D. & Guinard, J.-X. Consumer preferences for black coffee are spread over a wide range of brew strengths and extraction yields. *J. Food Sci.* **86**, 194–205 (2021). 33340109 10.1111/1750-3841.15561

[9] J. Hoffmann, *The world atlas of coffee*. (Hachette, 2018).

[10] Harwood, W. S., McLean, K. G., Ennis, J. M., Ennis, D. M. & Drake, M. Comparison of preference mapping with projective mapping for characterizing consumer perception of brewed black coffees. *J. Sens. Stud.* **35**, e12563 (2020).

[11] Lichters, M., Möslein, R., Sarstedt, M. & Scharf, A. Segmenting consumers based on sensory acceptance tests in sensory labs, immersive environments, and natural consumption settings. *Food Qual. Prefer.* **89**, 104138 (2021).

[12] Jaeger, S. R., Worch, T., Phelps, T., Jin, D. & Cardello, A. V. Preference segments among declared craft beer drinkers: perceptual, attitudinal and behavioral responses underlying craft-style vs. traditional-style flavor preferences. *Food Qual. Prefer.* **82**, 103884 (2020).

[13] van Kleef, E., van Trijp, H. C. M. & Luning, P. Internal versus external preference analysis: an exploratory study on end-user evaluation. *Food Qual. Prefer.* **17**, 387–399 (2006).

[14] Varela, P., Beltrán, J. & Fiszman, S. An alternative way to uncover drivers of coffee liking: Preference mapping based on consumers’ preference ranking and open comments. *Food Qual. Prefer.* **32**, 152–159 (2014).

[15] Bhumiratana, N., Wolf, M., Chambers IV, E. & Adhikari, K. Coffee drinking and emotions: are there key sensory drivers for emotions? *Beverages* **5**, 27 (2019).

[16] Guinard, J.-X. et al. A new coffee brewing control chart relating sensory properties and consumer liking to brew strength, extraction yield, and brew ratio. *J. Food Sci.* **88**, 2168–2177 (2023). 36988107 10.1111/1750-3841.16531

[17] Rothman, L. & Parker, M. J. Just-about-Right (JAR) Scales: Design, Usage, Benefits, and Risks. (ASTM International, 2009).

[18] Lawless, H. T. & Heymann, H. *Sensory Evaluation of Food: Principles and Practices*. (Springer Science & Business Media, 2010).

[19] Raschka, S. Model evaluation, model selection, and algorithm selection in machine learning. arXiv preprint arXiv:1811.12808 (2018).

[20] Frost, S. C., Ristenpart, W. D. & Guinard, J.-X. Effects of brew strength, brew yield, and roast on the sensory quality of drip brewed coffee. *J. Food Sci.* **85**, 2530–2543 (2020). 32652586 10.1111/1750-3841.15326

[21] Batali, M. E., Ristenpart, W. D. & Guinard, J.-X. Brew temperature, at fixed brew strength and extraction, has little impact on the sensory profile of drip brew coffee. *Sci. Rep.* **10**, 16450 (2020). 33020560 10.1038/s41598-020-73341-4 PMC7536440

[22] Batali, M. E., Frost, S. C., Lebrilla, C. B., Ristenpart, W. D. & Guinard, J.-X. Sensory and monosaccharide analysis of drip brew coffee fractions versus brewing time. *J. Sci. Food Agric.* **100**, 2953–2962 (2020). 32031262 10.1002/jsfa.10323

[23] Oestreich, J. S. Chemistry of coffee. Reference Modul In Chemistry, Molecular Sciences Engineering 1–28 (2010).

[24] Cordoba, N., Peterson, D. & Giuliano, P. “How Sweet Coffee Tastes! Towards an Understanding of Coffee Sweetness,” SCA News, vol. 25, no. 22, Oct. 2025, Accessed: Dec. 15, 2025. [Online]. Available: https://sca.coffee/sca-news/25/issue-22/understanding-coffee-sweetness.

[25] Ristenpart, W. D., Cotter, A. R. & Guinard, J.-X. Impact of beverage temperature on consumer preferences for black coffee. *Sci. Rep.* **12**, 20621 (2022). 36450773 10.1038/s41598-022-23904-4 PMC9712614

[26] Batali, M. E., Cotter, A. R., Frost, S. C., Ristenpart, W. D. & Guinard, J.-X. Titratable acidity, perceived sourness, and liking of acidity in drip brewed coffee. *ACS Food Sci. Technol.* **1**, 559–569 (2021).

[27] Barahona, I., Sanmiguel Jaimes, E. M. & Yang, J. Sensory attributes of coffee beverages and their relation to price and package information: a case study of Colombian customers’ preferences. *Food Sci. Nutr.* **8**, 1173–1186 (2020). 32148823 10.1002/fsn3.1404 PMC7020298

[28] Bichlmaier, C. et al. Contribution of mozambioside roasting products to coffee’s bitter taste. *Food Chem.* **469**, 142547 (2025). 39709917 10.1016/j.foodchem.2024.142547

[29] Liang, J., Batali, M. E., Routt, C., Ristenpart, W. D. & Guinard, J.-X. Sensory analysis of the flavor profile of full immersion hot, room temperature, and cold brewed coffee over time. *Sci. Rep.* **14**, 19298 (2024). 39164402 10.1038/s41598-024-69867-6 PMC11335879

[30] Peryam, D. R. & Girardot, N. F. Advanced taste-test method. *Food Eng.* **7**, 58–61 (1952).

[31] Peryam, D. R. & Pilgrim, F. J. Hedonic scale method of measuring food preferences. *Food Technol.* **11**, 9–14 (1957).

[32] Guyon, I., Weston, J., Barnhill, S. & Vapnik, V. Gene selection for cancer classification using support vector machines. *Mach. Learn.* **46**, 389–422 (2002).

[33] Platt, J. et al. Probabilistic outputs for support vector machines and comparisons to regularized likelihood methods. *Adv. Large Margin Classif.* **10**, 61–74 (1999).

[34] Zhang, H. The optimality of naive Bayes. *Aa* **1**, 3 (2004).

[35] Freund, Y., Schapire, R. & Abe, N. A short introduction to boosting. *J. -Jpn. Soc. Artif. Intell.* **14**, 1612 (1999).

[36] G. E. Hinton, “Connectionist learning procedures,” in *Machine Learning* (pp. 555–610) (Elsevier, 1990).

[37] Breiman, L. Random forests. *Mach. Learn.* **45**, 5–32 (2001).

[38] Liu, F. T., Ting, K. M. & Zhou, Z.-H. “Isolation Forest,” in *2008 Eighth IEEE International Conference on Data Mining*, (pp. 413–422) (IEEE, 2008).

[39] Breunig, M. M., Kriegel, H.-P., Ng, R. T. & Sander, J. “LOF: identifying density-based local outliers,” in *Proc. 2000 ACM SIGMOD International Conference on Management of Data*, in SIGMOD ’00. (pp. 93–104) (Association for Computing Machinery, 2000).

[40] Satopaa, V., Albrecht, J., Irwin, D. & Raghavan, B. “Finding a” kneedle” in a haystack: Detecting knee points in system behavior,” in *2011 31st International Conference On Distributed Computing Systems Workshops*, (pp. 166–171) (IEEE, 2011).

[41] Lloyd, S. Least squares quantization in PCM. *IEEE Trans. Inf. Theory* **28**, 129–137 (1982).

[42] Rousseeuw, P. J. Silhouettes: a graphical aid to the interpretation and validation of cluster analysis. *J. Comput. Appl. Math.* **20**, 53–65 (1987).

[43] Caliński, T. & Harabasz, J. A dendrite method for cluster analysis. *Commun. Stat.* **3**, 1–27 (1974).

[44] Schwarz, G. “Estimating the dimension of a model,” Ann. Stat. **6**, 461–464 (1978).

