# Supplementary material for: AI-driven prediction of consumer liking of coffee from sensory data
Source: NPJ Sci Food. 2026 Mar 14;10:142. doi: 10.1038/s41538-026-00779-7 (PMC13133105; doi:10.1038/s41538-026-00779-7)
Supplement: Supplementary file 1 — Supplementary materials [file 41538_2026_779_MOESM1_ESM.pdf]

AI prediction of consumer liking of coffee from sensory data

Michael Gunning[1,2,3], Maite Pilar Serantes Laforgue[4], Jean-Xavier Guinard[4,5], and Ilias Tagkopoulos[1,2,3*]

[1]*Department of Computer Science, University of California, Davis, Davis, California, 95616 United States*

[2]*Genome Center, University of California, Davis, Davis, California, 95616 United States*

[3]*USDA/NSF AI Institute for Next Generation Food Systems (AIFS), University of California, Davis, Davis, California, 95616 United States*

[4]*Department of Food Science and Technology, University of California, Davis, Davis, California, 95616 United States*

[5]*UC Davis Coffee Center, University of California, Davis, Davis, California, 95616 United States*

[*]Corresponding author: itagkopoulos@ucdavis.edu

Supplementary materials and results

1. Supplementary Figures

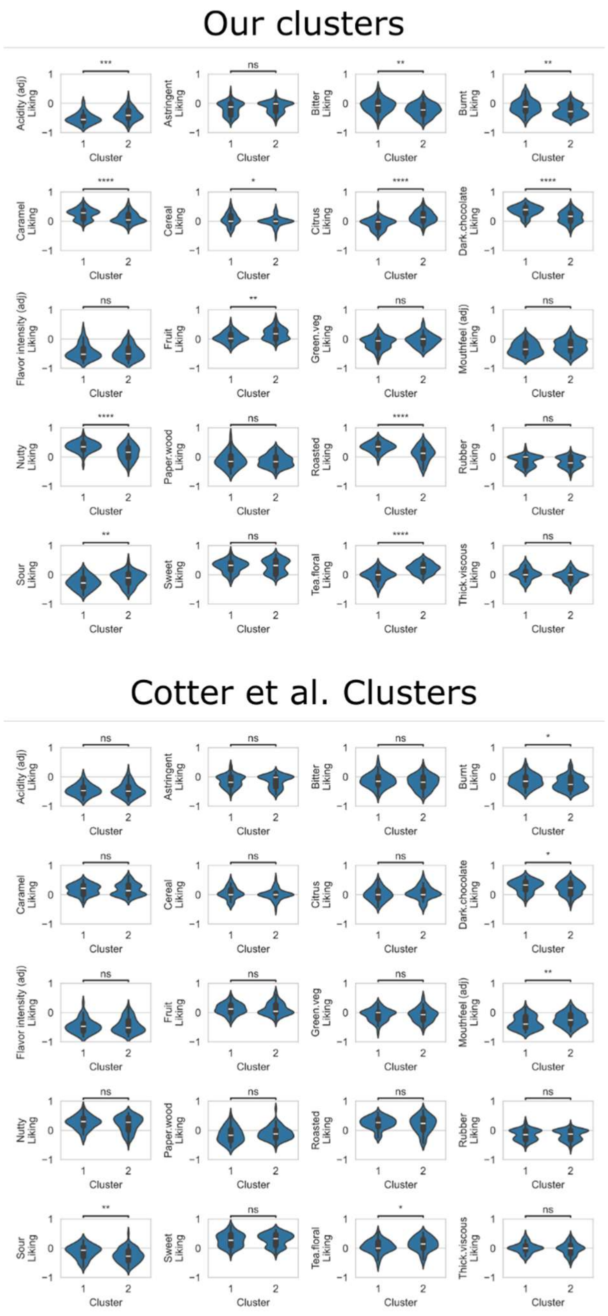

Supplementary Figure S1: Comparison of per-cluster liking for each sensory feature. Our clustering method achieves better separation of clusters in terms of sensory features, with 12 features having significantly different liking per cluster (Kruskal-Wallis test, pvalue <0.05) compared to Cotter et al. clusters which only identified 5 significantly different sensory preferences.

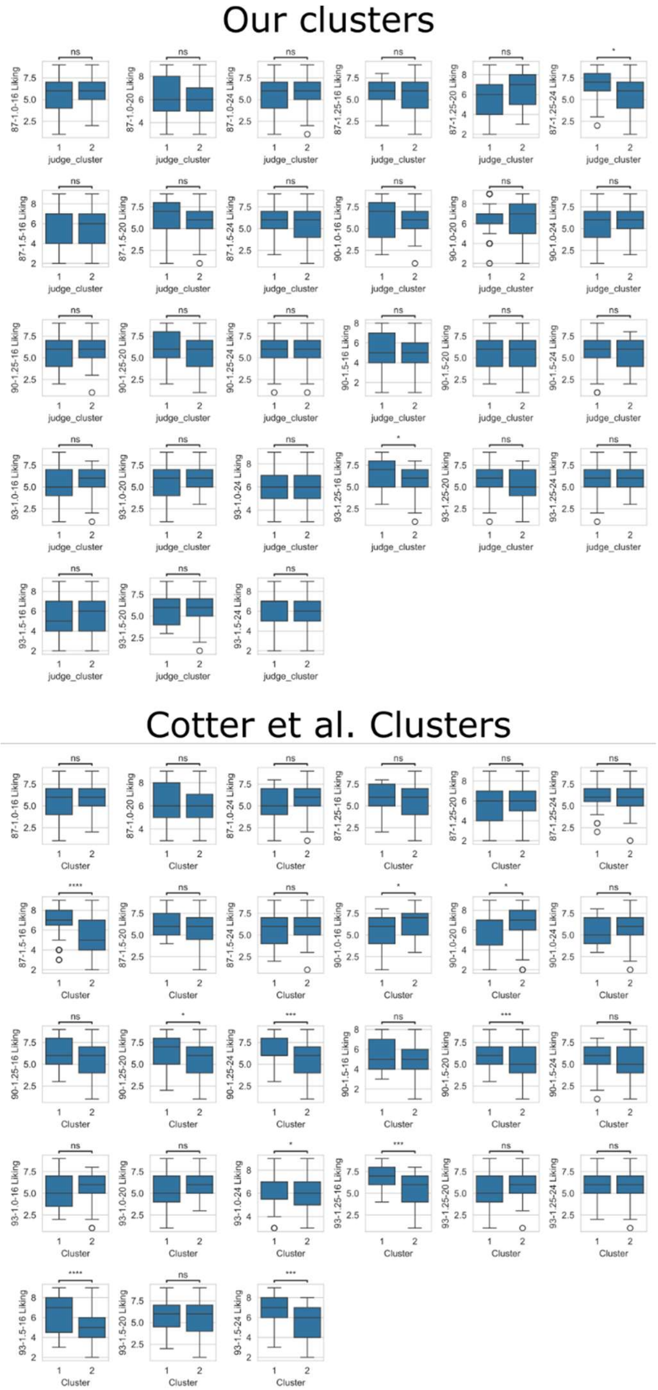

Supplementary Figure S2: Comparison of per-cluster liking for each coffee brew. Cotter et al. clustering achieves better separation of clusters in terms of preferred brews, with 10 brews having significantly different liking per cluster (Kruskal-Wallis test, pvalue <0.05) compared to our clustering method which only identified 2 significantly different brews.

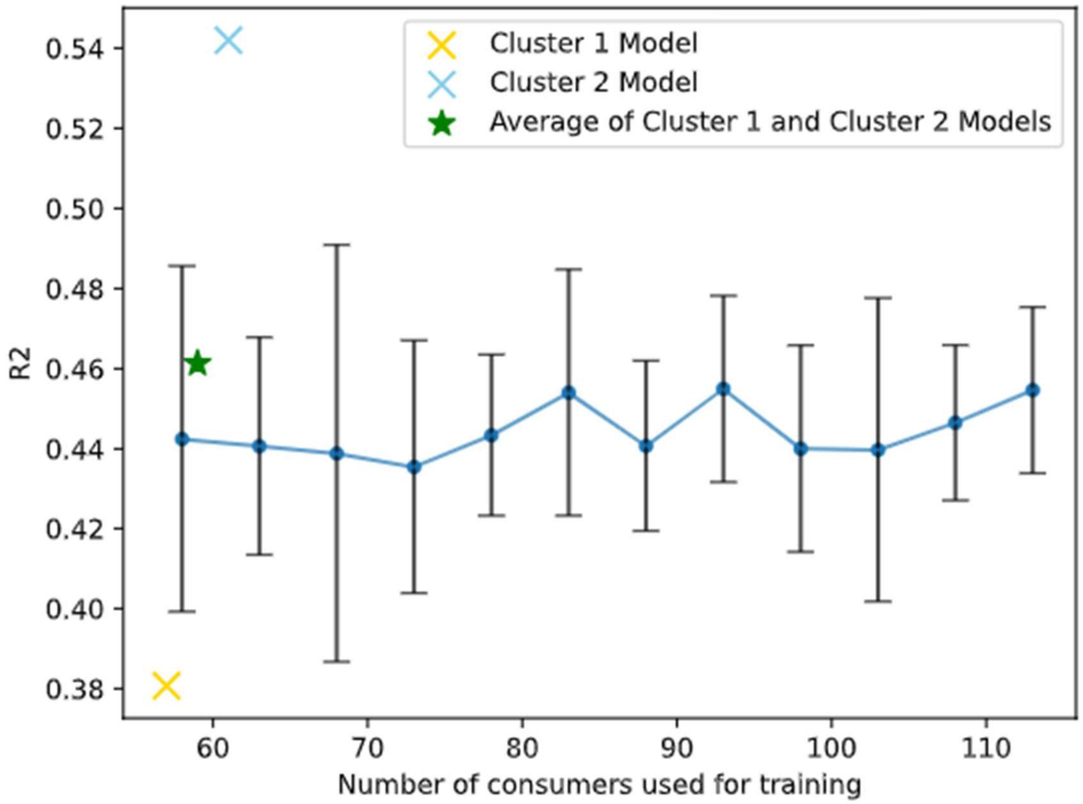

Supplementary Figure S3: Analysis of model performance with sequentially more arbitrary consumers removed from dataset. Each point is the average of 10 trials of randomly selected judges removed. Error bars are the standard deviation of each set of 10 trials. Performance of models trained on each cluster are compared, as these were both trained on a subset of n=61 and n=57 consumers, making for a fairer comparison than a model trained with all 118 consumers.

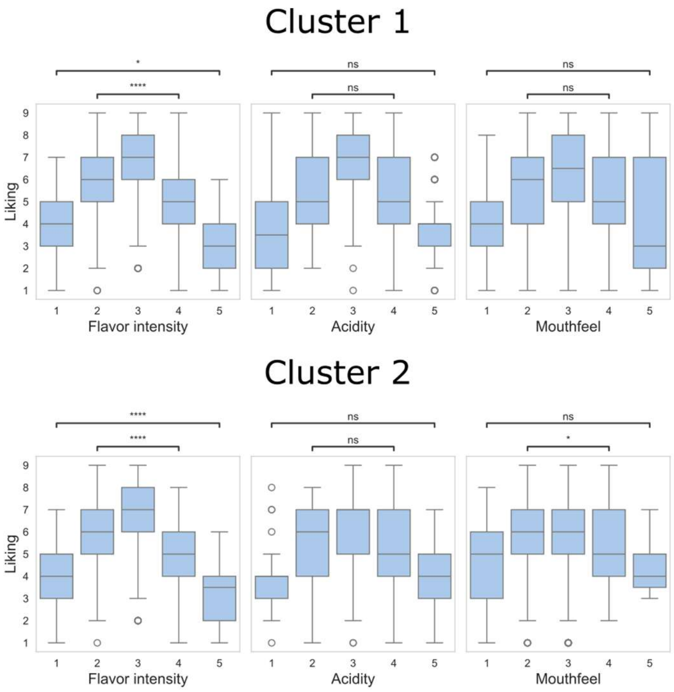

Supplementary Figure S4: Per-cluster JAR feature liking. Although there are some differences between clusters for Acidity and Mouthfeel, in general per-cluster JAR liking was similar to the JAR liking of all judges, with significant differences in liking for too much vs too little flavor intensity.

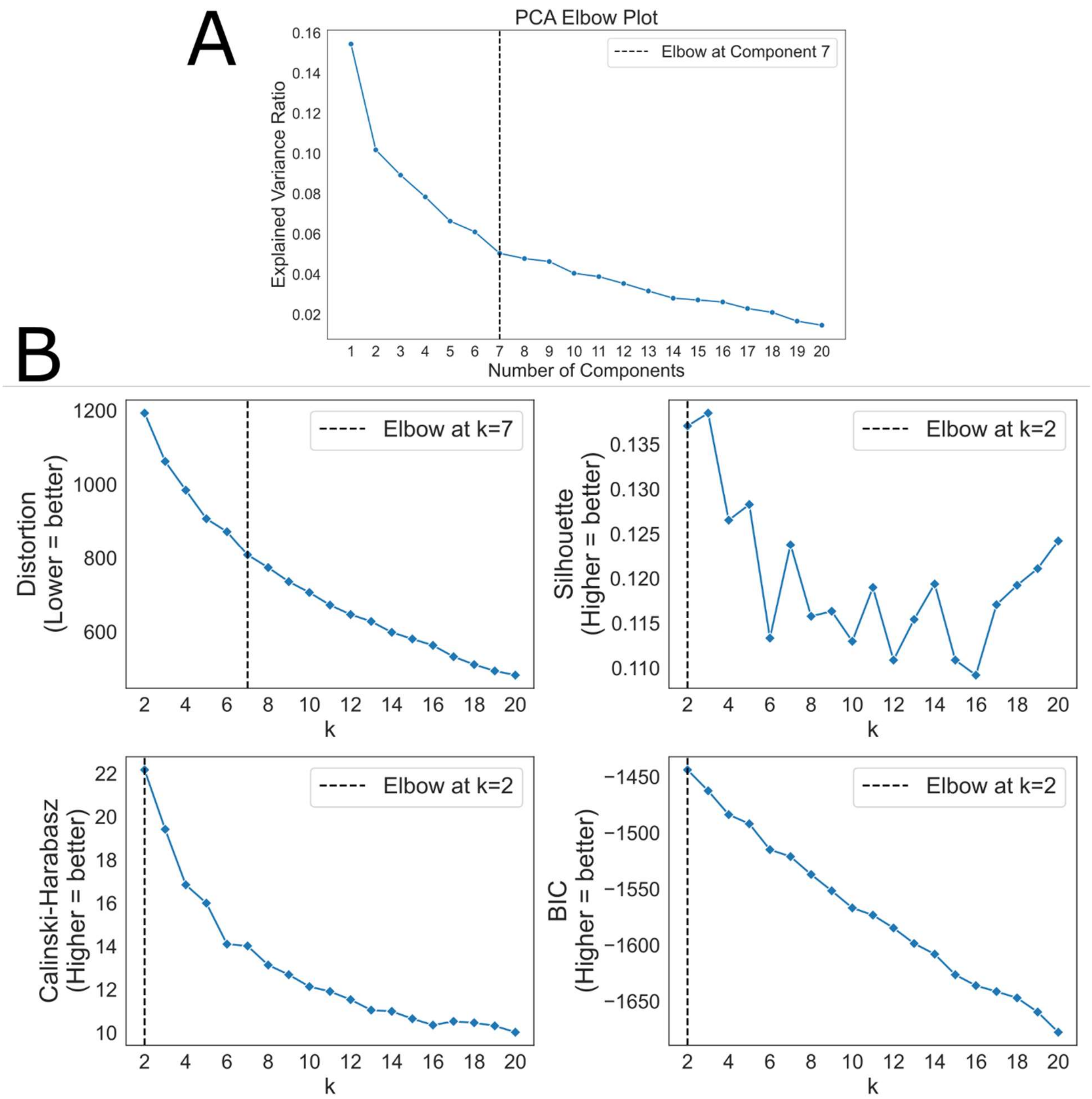

Supplementary Figure S5: A. PCA scree plot for pre-clustering dimensionality reduction. B. Selection of k by elbow method using 4 metrics: distortion score, silhouette score, Calinski-Harabasz score, and BIC. Although not all metrics agreed with k=2, in general lower numbers of clusters had higher scores. Additionally, selecting a k=2 makes our results more comparable with the results from Cotter et al.
